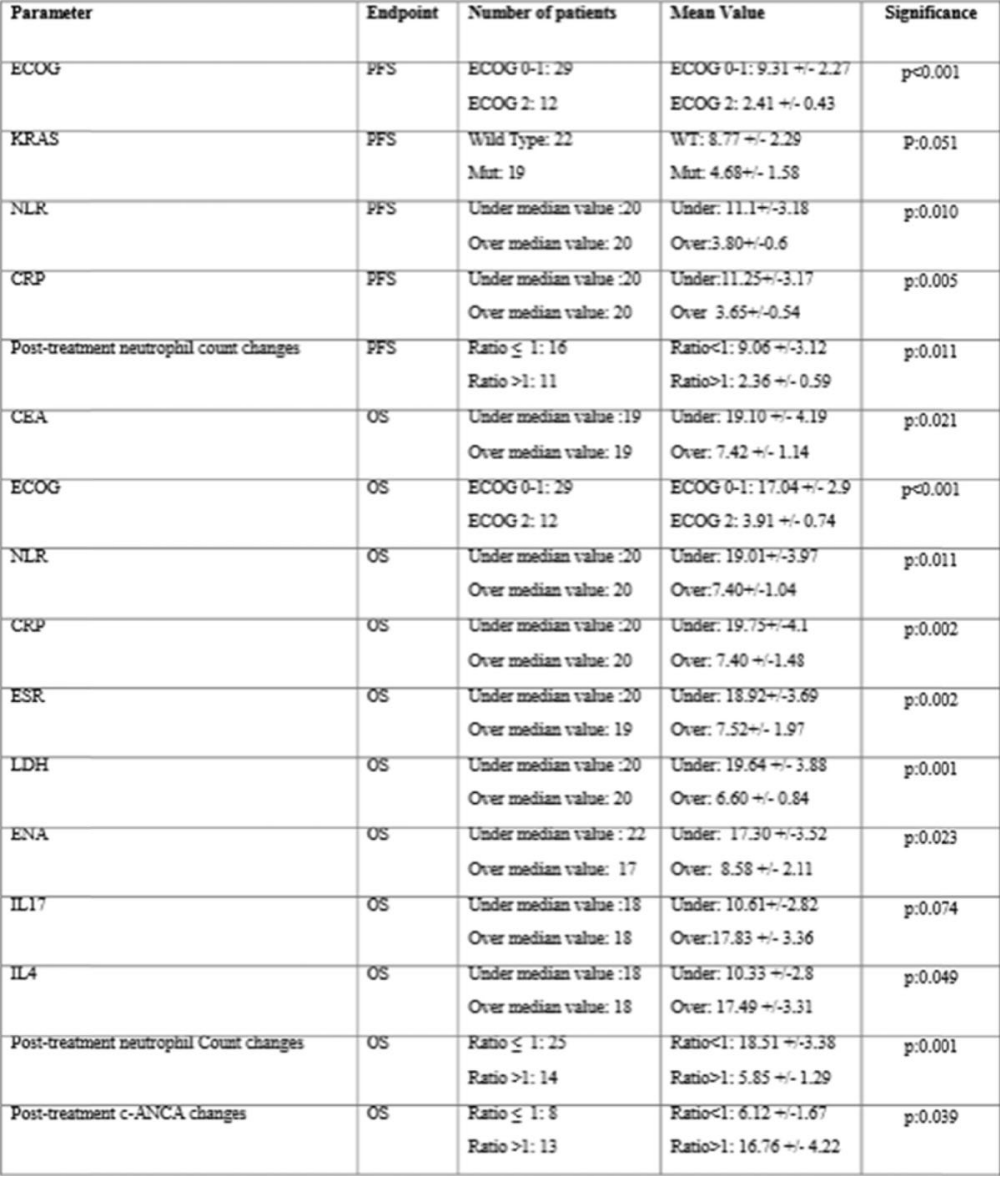# Inflammatory status affects the antitumor activity of poly-epitope-peptide vaccination against the thymidylate synthase in metastatic colo-rectal cancer patients enrolled in TSPP/VAC-1 Phase Ib trial

**DOI:** 10.1186/2051-1426-3-S2-P443

**Published:** 2015-11-04

**Authors:** Pierpaolo Correale, Valerio Nardone, Cirino Botta, Elodia Martino, Pierpaolo Pastina, Cristina Ulivieri, Maria Grazia Rossetti, Antonella Fioravanti, Claudia Gandolfo, Francesco Carbone, Tatiana Cosima Baldari, Pierosandro Tagliaferri, Luigi Pirtoli, Maria Grazia Cusi

**Affiliations:** 1 Unit of Radiotherapy and Unit of Radiology, Department of Oncology, Siena, Italy; 2 Department of Experimental and Clinical Medicine, “Magna Graecia” University and Medical Oncology Unit, Fondazione Tommaso Campanella”, Catanzaro, Italy; 3 Department of Life Sciences, Siena University, Siena, Italy; 4 Unit of Pharmacy, Siena University Hospital, Siena, Italy; 5 Unit of Rheumatology, Department of Clinical Medicine and Immunologic Sciences, Siena, Italy; 6 Microbiology Unit, Department of Medical Biotechnology, Siena, Italy; 7 Unit of Radiology, Siena Hospital, Siena, Italy

## 

Thymidylate synthase (TS) is a tumor-associated-enzyme crucial for DNA replication and inhibited by 5′-fluorouracil. TSPP is a previously characterized anticancer poly-epitope peptide vaccine to TS (*Correale P, JNCI 2005 97:1437*). TSPP/VAC-1 is a three-arm dose-finding Phase-Ib trial aimed to test in pretreated-advanced cancer patients, TSPP-vaccination alone (arm A), together with GM-CSF and low dose Aldesleukine (arm B), or together with chemo-immunotherapy according to the GOLFIG regimen (*Correale P, JCO*, *2005, 23:8950*) (arm C). TSPP resulted safe, its MTD was not achieved, while its most-effective-biological-dose was 300µg. As the most promising antitumor effects of TSPP were observed in colo-rectal cancer (mCRC) patients (*Cusi MG, CIIT, 2015, epub*), we decided of carrying-out a new study to evaluate in this subset of patients, the potential ability of a predefined panel of markers to predict their antitumor response to TSPP. We thus evaluated41 mCRC patients, 20 males and 21 females, with a good performance status, enrolled between May 2011 and Jan 2013. Our parameters were correlated with progression free survival (PFS) and overall survival (OS) by performing a Kaplan Meier analysis. The baseline marker values were divided in two groups according to their median values, while the changes relative to baseline values (post-treatment values) were divided according to a fold ratio ≤ or >1. Patients' PFS and OS were 6.9 and 11.3 months, respectively; there were no significant differences in PFS and OS correlated with treatment arm (A vs. B vs. C), number and type of previous treatments, sex, age, TS expression, HLA2.1 haplotype or expression of peripheral CTLs, regulatory-T cells, central- and effector-memory-T cells. Patients bearing K-ras mutations, showed a trend to a shorter PFS (p:0.051) and no differences in OS (p=0.16). Patients' outcome was instead, inversely correlated with performance status (ECOG 0-1 vs. 2; PFS, p1, OS, p:0.039). These results suggest that inflammatory status and autoimmunity may affect TSPP antitumor activity in mCRC patients. These results deserve to be considered for the design of new studies.

**Figure 1:**